# Droplet Sizes Emitted from Demonstration Electric Toothbrushes

**DOI:** 10.3390/ijerph18052320

**Published:** 2021-02-26

**Authors:** Erwin P. Mark, Michael A. O. Lewis, Filippo Graziani, Boris Atlas, Joern Utsch

**Affiliations:** 1 Research & Development, Procter & Gamble Service GmbH, German Innovation Center, Frankfurter Straße 145, 61476 Kronberg, Germany; mark.e@pg.com (E.P.M.); utsch.j@pg.com (J.U.); 2 School of Dentistry, Cardiff University, Cardiff, Wales CF14 4XY, UK; lewismao@cardiff.ac.uk; 3 Department of Surgical, Medical and Molecular Pathology and Critical Care Medicine, University of Pisa, 56126 Pisa, Italy; filippo.graziani@med.unipi.it; 4 Sub-Unit of Periodontology, Halitosis and Periodontal Medicine, University Hospital of Pisa, 56126 Pisa, Italy

**Keywords:** toothbrushing, electric toothbrush, aerosols, oral hygiene

## Abstract

The COVID-19 pandemic has drawn attention to microbial transmission risk via aerosols in dental practice. Demonstration electric toothbrushes are used intra-orally for education. The aim of this investigation was to measure the size of droplets emitted by the brush head of two demonstration oscillating-rotating electric toothbrushes. Measurement of droplet production and size was recorded in vitro using three methods: (1) Malvern Spraytec (LASER particle size measurement device with detectable particle size of 0.1–2500 µm) and brushes mounted on a 3D-printed, two-shell form-fit fixture with a supply of tap water; (2) a DustTrak aerosol measurement device and toothpaste slurry, with brushing simulated in the oral cavity of a phantom head; (3) high-speed visualization in a simulated-use situation in the oral cavity of a phantom head, with individual evaluation of tap water, water with detergent, 70% ethanol, glycerin and toothpaste slurry. Both brushes showed the size of emitted droplets was consistently between 200 and 1200 µm, categorized as splatter (dental aerosols are <50 µm diameter). No significant incremental aerosol-sized matter was detected during toothbrush operation. The high-speed video visualization confirmed only splatter-sized droplets during operation. These findings indicate that oscillating-rotating toothbrushes do not produce aerosol-sized particles during simulated use.

## 1. Introduction

Professional preventive dental care is an important component of the maintenance of oral health for many individuals. During these preventive visits, as well as during those necessary for treatment, patients usually receive guidance on oral hygiene procedures and recommendations for their self-care at home. Regular oral hygiene, reinforced during professionally directed education, can improve periodontal outcomes [1].

A contemporary form of oral hygiene advice includes the development of a novel approach using demonstration electric rechargeable toothbrushes to deliver interactive oral hygiene guidance. Dental professionals utilize a demonstration rechargeable handle, brush heads and disposable sheaths, along with a protocol for providing hygienic electric toothbrushing advice to patients [2]. This procedure introduces patients to the oscillating-rotating electric toothbrush technology, which has been clinically proven to deliver superior plaque and gingival health outcomes versus manual toothbrushes [3,4,5].

The recommended protocol builds on the standard practice associated with various types of instrumentation used in the dental clinic and an understanding of routes of infection transmission between people [6,7,8,9]. The emergence of the COVID-19 pandemic has resulted in a need to determine whether the use of electric toothbrushes may represent a potential risk due to aerosol particle generation, defined as <50 µm [10,11]. This concern has been magnified due to outbreaks of previous respiratory diseases and those connected with the current global pandemic [12].

To date, it has been recognized that electric toothbrushes can produce splatter, defined as droplets >50 µm [10,11], consisting of a homogenous mixture of saliva, plaque and blood, in addition to toothpaste. However, there is no published information on the precise size and dynamics of the droplets produced when electric toothbrushes are in operation. Since aerosols are generally accepted to represent an essential component of infection control in dentistry [13], the aim of this investigation was to measure the size and dynamics of particles that are produced when the electric toothbrushes included in the unique oral hygiene demonstration program are used in the dental clinic setting.

## 2. Materials and Methods

The scope of the investigation was to measure the size of liquid droplets emitted by the moving part of the brush head of two oscillating-rotating toothbrushes: (1) Oral-B iO with an Ultimate Clean brush head (TB1—Procter & Gamble, Cincinnati, OH, USA); (2) Oral-B Genius with a Cross-Action brush head (TB2, Procter & Gamble, Cincinnati, OH, USA).

**Figure 1:**
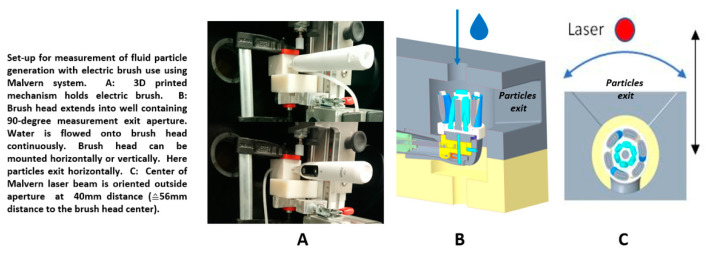
Schematic of Malvern system experimental setup.

**Figure 2:**
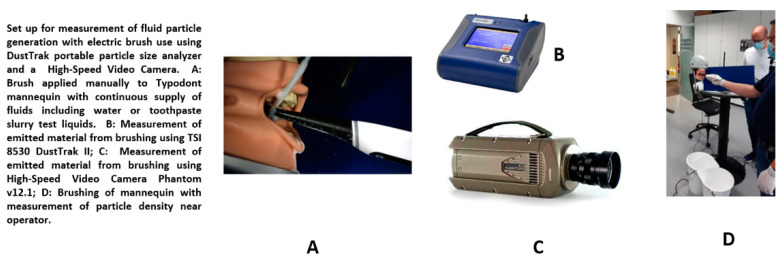
Schematic of DustTrak and High-Speed Video Camera experimental setups.

The materials and methods applied to the investigation––Malvern Spraytek, DustTrak, and a high-speed video camera system––were chosen because they are specifically designed to address the unique requirements for spray characterization and deliver robust, reproducible droplet size data (Figure 1 and Figure 2).

### 2.1. Test 1: Malvern Spraytec

The first test regime measured droplet formation with use of a Malvern Spraytec (Malvern Panalytical Ltd., Malvern, UK) calibrated LASER particle size measurement device with a detectable particle size of 0.1–2500 µm. A jig was constructed, allowing brushes to be mounted on a 3D-printed, 2-shell form-fit fixture, as shown in Figure 1A. An aperture was created around the brush head, with a 90-degree opening to permit developed droplets to exit the brush head. The laser detector was positioned 40 mm beyond the aperture. The brush heads were tested for particle generation in both horizontal (TB1 and TB2) and vertical (TB1) orientations of the oscillating-rotating motion. Water was run over the brush heads at 60 mL/minute to provide liquid for adequate particle generation from the wet brushes. Data were collected for 30 s once the electric toothbrushes were switched on. Data were displayed as histograms of generated particles, and distribution volume fractions of generated droplets were developed. As a reference standard, a spray of a disinfectant (isopropyl alcohol 75% *v/v*, glycerol 1.45% *v/v*, hydrogen peroxide 0.125% *v/v*; World Health Organization Handrub Formulation 2) was generated by a generic pump spray applicator to compare droplet size distributions and confirm sensitivity of the system.

### 2.2. Test 2: DustTrak

Separately, a DustTrak aerosol monitor was used to assess particle emission during simulated brushing in the oral cavity of a mannequin phantom head (which was fitted with a full permanent dentition typodont), with test brushes applied by an operator. This setup simulated brushing patients’ teeth by (a) a dental professional during oral hygiene advice and demonstration of an oscillating-rotating electric toothbrush and (b) brushing by a caregiver. Test liquids, including tap water, water with detergent, 70% ethanol, glycerin and toothpaste slurry, were run into the oral cavity of the mannequin at 60 mL/minute, and the dentition was thoroughly brushed by the operator (Figure 2D). Emitted particles were measured using a DustTrak portable particle analyzer (DustTrak™ DRX Aerosol Monitor 8530). In the first experiment, the DustTrak suction inlet was positioned next to the mannequin’s oral cavity. In the second experiment, it was positioned right next to the operator’s face, measuring the potential inhaling uptake of said operator. The DustTrak exhibits sensitivity to measure particle sizes ranging from 0.1 to 10 µm.

### 2.3. Test 3: High-Speed Video Camera Imaging

The high-speed video camera (Phantom v12.1—Vision Research Inc, Wayne, NJ, USA) was positioned to capture emitted particles when brushing a denture in the oral cavity of a mannequin phantom head. Matlab software was used to calculate the flight distance of emitted particles captured by the high-speed video camera based on the trajectory of emitted particles, treating them in a ballistic manner. Ballistic flight range was calculated using maximum detected speed from video with different drop sizes (1.0, 0.3, 0.1 mm) and ejection angles (horizontal/45°). Results with air friction were based on a spherical droplet shape. A reference standard spray of disinfectant was again generated by a generic pump spray applicator to compare droplet size distribution and visualization.

## 3. Results

### 3.1. Test 1: Malvern Spraytec

**Figure 3:**
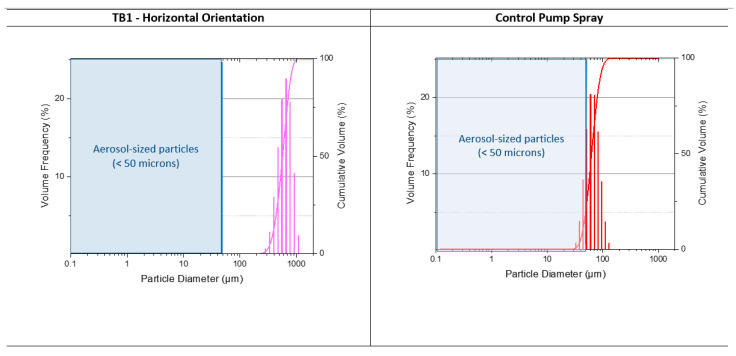
Particle size distribution of emitted particles from TB1 (horizontal orientation) and control spray—Malvern analysis.

The Malvern particle analysis showed that droplet sizes for the test toothbrushes in horizontal and vertical orientation averaged roughly 0.5 mm (500 µm), as seen in Table 1 and Figure 3, which is in the range associated with splatter. No particles smaller than 200 µm were detected. The reference standard illustrated the sensitivity of the laser for measurement of droplets of <50 µm. Histogram distributions of emitted particles from reference standard spray and TB1 horizontal orientation are displayed in Figure 3.

### 3.2. Test 2: DustTrak

**Figure 4:**
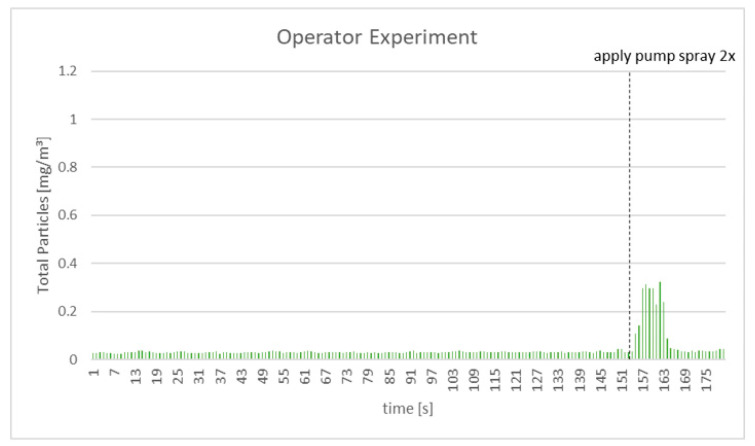
DustTrak measurements of particles generated at operator’s distance when brushing a mannequin’s oral cavity (see Figure 2D) for 2 min with a slurry and then applying the reference spray.

The DustTrak evaluation showed that virtually no emitted particles were observed for all test liquids during operator brushing of the test mannequin, as seen in Supplementary Table S1 and illustrated in Figure 4. Similar results were observed for both test brushes. At the end of test mannequin brushings, reference disinfectant was sprayed in the field of the DustTrak, with clear instrumental pick-up of the material, again illustrating the sensitivity of the operational setup for detection of potentially emitted particles from brushing.

### 3.3. Test 3: High-Speed Video Camera

**Figure 5:**
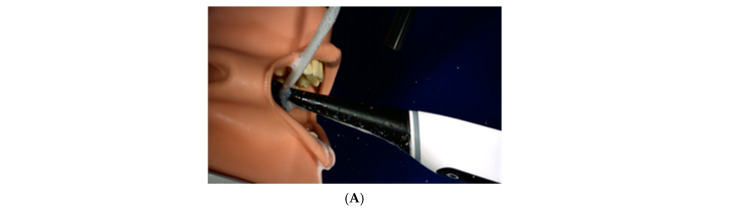

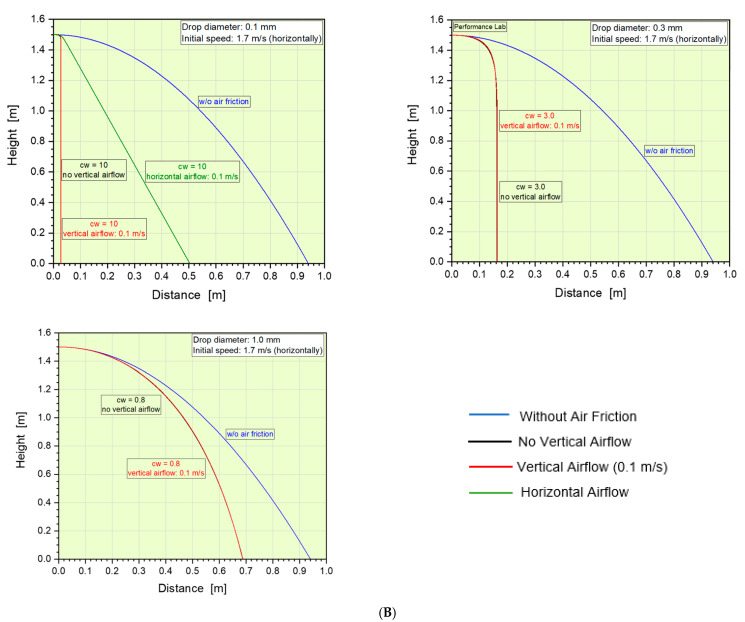
(**A**) High-speed video (still image) of particle emission with operator brushing mannequin. (**B**) Visualization of droplet trajectories of various droplet sizes.

Findings from the high-speed video camera (with videos of the brushing included as Supplementary Material, Video S1 and Video S2) also showed droplet formation during brushing, representing the phenomena of splatter and corroborating the data analyzed by the Malvern. A still image of particles emitted during operator brushing of the mannequin is shown in Figure 5A, which captures the large size of the droplets generated. The trajectory analyses of emitted drops from the brush (Figure 5B; Table 2) show the splatter generated by operator brushing was not projected to travel more than 95 cm in distance from the location of the patient. Time for the droplets to fall from a drop height of 150 cm was calculated for a range of droplet sizes (See Table 2). A falling time of <1 s was reported for droplet sizes of 1 mm or greater in typical indoor conditions. Of the recorded droplets, 99% were larger than 0.3 mm and had a time to fall down *t* < 1.8 s. Maximum times for droplets to fall down were calculated as *t* < 7 s for droplet sizes of 0.1 mm (droplets of this size were not observed in this experiment) in extreme conditions of upward airflow of 0.1 m/s (assuming convection current impacts of air-conditioning systems).

## 4. Discussion

This in vitro research revealed that oscillating-rotating electric toothbrushes produce few droplets when used for demonstration purposes with various liquids. Droplets that were emitted were consistently between 200–1200 µm, with the average size around 500 µm, and settled within 1 m of their source. A total of 99% of all recorded droplets fell in less than 1.8 s. These droplet sizes can be categorized as “splatter”, and no significant incremental aerosol-sized particles were detected during toothbrush operation in any of the protocols. Control measures of volatized spray from a spray bottle validated instrumental sensitivity to detect aerosols under conditions of testing.

Collectively, these findings indicate that the demonstration electric toothbrushes generate splatter, but, importantly, do not emit aerosol-sized particles when being used. It is essential to recognize the difference between “splatter” and “aerosol”. Splatter is defined as airborne particles larger than 50 μm in diameter [10,11]. Micik and colleagues stated that these large droplets behave in a ballistic manner [10,11]. After forcible ejection from the operational site, they follow an arc and trajectory similar to that of a bullet, and upon descent, these droplets fall to the floor or nearby surfaces. Crucially, particles and droplets of this size are too large to become suspended in the air. Hence, it was postulated that splatter is relatively easy to manage in a clinical setting. In contrast, aerosols are defined as particles less than 50 μm in diameter. Particles of this size, and particularly fine aerosols of 0.5–10 µm, have been described as small enough to stay airborne for extended periods prior to settling on environmental surfaces. While airborne, aerosol particles can, in principle, pass through face masks and therefore have the potential to enter the respiratory tract [14,15,16]. Researchers have postulated that the greatest airborne infection threat in dentistry comes from aerosols (particles less than 50 μm in diameter) [14,15].

Dental instruments such as dental handpieces, ultrasonic scalers, air polishers and air abrasion units are known to produce splatter in their operation and in some cases also generate aerosols [13]. However, the results reported here confirm that the demonstration electric toothbrushes tested do not generate aerosol. At the present time there are only two demonstration electric toothbrushes available that are specifically designed for use in the dental surgery as an adjunct to the practical delivery of oral hygiene instruction. Both these devices were evaluated, and the rationale of the study was to understand an eventual aerosol production within the dental practice, as opposed to what would be produced at home where the patient brushes. Therefore these findings confirm such devices can be used safely, without risk of aerosolization, in the clinical setting as part of the effective delivery of oral hygiene advice, using the recommended protocol [2]. The demonstration toothbrushes are used in dental clinics under universal infection control conditions. In this respect, any indirect transfer involving hands, surfaces and other equipment is addressed by the routine infection prevention protocols and procedures. The primary aim of the research was to determine the nature of any aerosol or splatter that was generated by mechanical toothbrushes. The results demonstrated that no aerosol- sized particles that could potentially remain within environment were produced. Minimal splatter was observed, but these droplets would fall within the immediate controlled area of the patient. These findings were reassuring from an infection and safety perspective. However, they do not necessarily mean the risk of virus/bacteria transmission is reduced, as standard disinfection procedures must be followed to ensure droplets are cleaned from surfaces. Furthermore, these data cannot necessarily be applied to other electric toothbrush technologies, and additional research would be required to characterize the spray that they may produce during operation.

## 5. Conclusions

This research revealed that the use of the oscillating-rotating electric toothbrushes tested produces splatter droplets and not aerosol-sized particles. Therefore, these toothbrushes can be used safely, without risk of aerosolization, in dental clinics by trained dental professionals for intra-oral demonstration purposes with patients, using standard infection control procedures.

## Figures and Tables

**Table 1:** Distribution statistics for TB1, TB2 and control spray.

	Dv(10)	Dv(50)	Dv(90)	Droplet Size Detected
Control spray	43.89 (μm)	62.92 (μm)	90.41 (μm)	25 to 185 µm
TB1—Horizontal	351.3 (μm)	536.9 (μm)	778.8 (μm)	≥200 µm
TB1—Vertical	382.8 (μm)	568.9 (μm)	791.3 (μm)	≥200 µm
TB2—Horizontal	384.2 (μm)	562.3 (μm)	782.9 (μm)	≥200 µm

Note: Dv(XX) refers to the volume of particles of drop size in percentiles. Dv(10): 10% of the particles are smaller than this value, 90% are bigger. Dv(50): 50% of the particles are smaller than this value, 50% are bigger—median value. Dv(90): 90% of the particles are smaller than this value, 10% are bigger.

**Table 2:** Droplet trajectories for various droplet sizes *.

Droplet Diameter	Height (m)	Time for Falling (s)		
		Without Air Friction	No Vertical Airflow	Vertical Airflow (0.1 m/s)
0.1 mm	1.0	0.32	1.61	2.35
	0.5	0.46	3.19	4.65
	0.0	0.56	4.76	6.95
CW			10	10
0.3 mm	1.0	0.32	0.59	0.65
	0.5	0.46	1.09	1.20
	0.0	0.56	1.59	1.76
CW			3.0	3.0
1.0 mm	1.0	0.32	0.35	0.36
	0.5	0.46	0.53	0.54
	0.0	0.56	0.68	0.69
CW			0.8	0.8

* Initial speed of 1.7 m/s (horizontally). CW = Drag coefficient.

## Data Availability

The data presented in this study are available on request from the corresponding author.

## Supplementary Materials

The following are available online at https://www.mdpi.com/1660-4601/18/5/2320/s1, Video S1: Water Droplet Formation with Brushing, Front View; Video S2: Water Droplet Formation with Brushing, Side View; Table S1: DustTrak Data.
